# Synchronous multiple unilateral parotid gland tumors of benign and malignant histological types: case report and literature review^☆^

**DOI:** 10.1016/j.bjorl.2016.03.002

**Published:** 2016-04-22

**Authors:** Aleksandra Ochal-Choińska, Antoni Bruzgielewicz, Ewa Osuch-Wójcikiewicz

**Affiliations:** Medical University of Warsaw, Department of Otolaryngology, Warsaw, Poland

## Introduction

Salivary gland tumors are a very heterogeneous group of lesions. According to the histological classification published by WHO in 2005, 10 types of benign and 24 types of malignant tumors of the salivary glands can be distinguished. All of these lesions are relatively rare, and represent only 3–4% of head and neck tumors. 80% of them are located within the parotid gland, and they usually present as a single parotid lesion.^1^ Unilateral parotid neoplasms are very uncommon, and they usually have the same histological type. The most common type of such a lesion is Warthin tumor (6–12% of all adenolymphomas).^2^ The coexistence of tumors of different histological types in the same parotid gland constitutes less than 0.3% of all salivary gland neoplasms.^3^ The most common histological combination is Warthin tumor and pleomorphic adenoma. Two different unilateral parotid neoplasms can be metachronous or synchronous. However, even when more than one tumor occur at the same time, they must be distinguished from hybrid tumors, in which we can find two or more distinct histological types of neoplasms having an identical origin in the same tissue.^4^

Synchronous benign and malignant ipsilateral parotid tumors are an extremely rare phenomenon. It was described for the first time by Tanaka in 1953 as mucoepidermoid carcinoma associated with Warthin tumor.^5^ To our knowledge no case in the literature mentioned the occurrence of a tumor consisting of carcinoma ex pleomorphic adenoma and Warthin tumor, which is reported in the present paper.

## Case report

**Figure 1:**
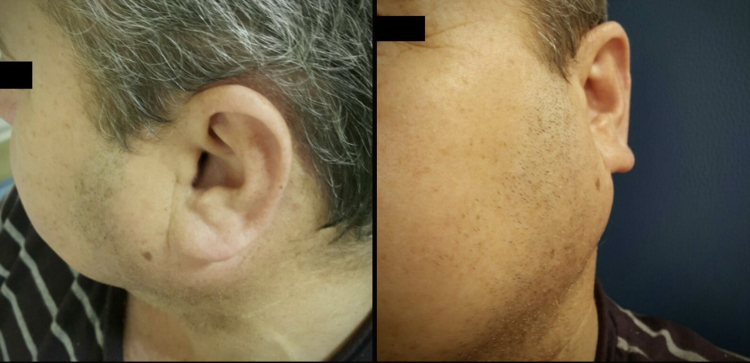
61-Year old patient with left parotid mass, on admission to the hospital.

61 year-old man presented with a 10 year history of a nontender, growing, left parotid mass. On clinical examination a firm, mobile mass was evident, with no overlying skin changes. The diameter of the lesion was 4 cm. No facial nerve palsy or lymphadenopathy was detected (Fig. 1).

**Figure 2:**
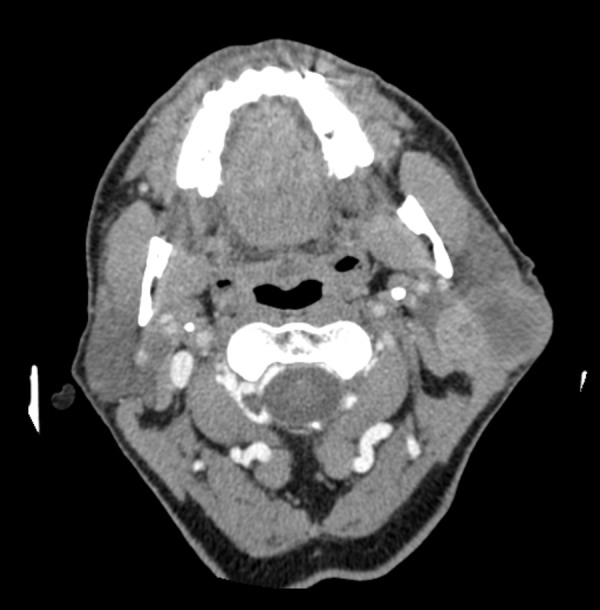
An enhanced CT scan of the parotid gland. Heterogeneously enhanced, left parotid mass, involving both lobes of the gland.

**Figure 3:**
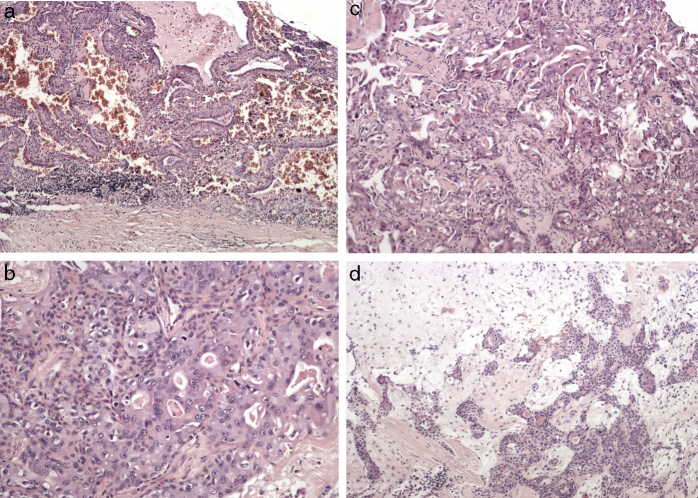
The histopatology of the resected specimen showing (A) Warthin tumor composed of lymphoid and epithelial cells, (B, C) malignant epithelial cells of carcinoma ex pleomorphic adenoma, in the background of (D) pleomorphic adenoma.

A CT scan revealed a 57 mm × 40 mm × 27 mm heterogeneously enhanced left parotid mass, involving both lobes of the gland (Fig. 2). There was no evidence of osteolytic changes in mandibular bones. Moreover, no invasions of the masseter muscle or of the parapharyngeal space were showed. A few 15–19 mm lymph nodes in the retro- and submandibular space were described. Fine needle aspiration biopsy confirmed pleomorphic adenoma. The patient underwent surgical treatment involving left subtotal parotidectomy. En bloc removal of the tumor was achieved with the excision of the superficial lobe. The facial nerve was preserved. Macroscopically, the mass was enveloped, solid and yellowish, with one cyst (1.5 cm in diameter) on the marginal part. Microscopically, the tumor mass included three different morphological patterns (Fig. 3). Pleomorphic adenoma was the dominant component. Within its tissue atypical cells of carcinoma ex pleomorphic adenoma were found. The marginal part of the solid mass included Warthin tumor cells. Surgical margins were free from neoplasm.

Due to the postoperative histopathological finding of malignant components of the tumor the patient was proposed a re-operation, involving the removal of the deep lobe of the parotid gland and elective neck dissection of I and II cervical lymph node groups. The patient did not give consent to surgical re-treatment. Therefore, even though CT scans did not indicate the presence of positive lymph nodes, the patient was referred for radiation treatment. No signs of recurrence were revealed during a follow-up after 5 years.

## Discussion

Multiple salivary gland tumors are occasionally seen, and account for 1.7–5% of parotid lesions. The vast majority of this phenomenon belongs to the same histological type of tumors, with Warthin tumor being the most common. Multifocal pleomorphic adenoma occurs rather infrequently.^6^, ^7^ Synchronous parotid tumors of different histology account for less than 0.3% of all salivary gland neoplasms. The most common combination is Warthin tumor and pleomorphic adenoma.^3^ Benign and malignant tumors in the ipsilateral parotid gland are extremely rare. Since Tanaka first reported the case of coexisting bilateral Warthin tumor and mucoepidermoid carcinoma, only 25 papers have reported the incidence of synchronous unilateral tumors of the parotid or periparotid region.^5^

According to previous reports, this type of lesions was more commonly observed in male patients, with the male-to-female ratio 1.3:1. The median patient's age was 66 years, and the average age – 64.1 years, which is almost 1 decade later than the incidence of malignancies in salivary glands in general.^1^ Warthin tumor was the most commonly described benign neoplasm (22 of 38 cases), pleomorphic adenoma was described less frequently (11 of 38 cases). There were solitary cases of other benign tumors, such as sebaceous lymphadenoma, oncocytoma and myoepithelioma. The most frequently observed malignant component was mucoepidermoid carcinoma (11 of 38 cases) and acinic cell carcinoma (8 of 38 cases). Hence, the most popular histological combination of neoplasms was Warthin tumor and mucoepidermoid carcinoma (9 of 38 cases) (Table 1).^3^, ^5^, ^6^, ^7^, ^8^, ^9^, ^10^, ^11^, ^12^, ^13^, ^14^, ^15^, ^16^, ^17^, ^18^, ^19^, ^20^, ^21^, ^22^, ^23^, ^24^, ^25^, ^26^, ^27^, ^28^

**Table 1:** Summary of papers concerning synchronous benign and malignant unilateral parotid gland tumors.

Author	Year	Benign tumor	Malignant tumor	Age	Gender
Our case	2015	Warthin tumor	Carcinoma ex pleomorphic adenoma	61	M
Jin J^8^	2011	Pleomorphic adenoma	Lymphoepithelial carcinoma	ND	ND
Srivastava S^9^	2009	Warthin tumor	Mucoepidermoid carcinoma	52	M
Roh JL^10^	2007	Warthin tumor	Adenocarcinoma	71	M
Tanaka S^11^	2007	Warthin tumor + pleomorphic adenoma	Salivary duct carcinoma	67	M
Ethunandan M^6^	2006	Warthin tumor	Acinic cell carcinoma	ND	ND
Bień S^12^	2006	Pleomorphic adenoma	Adenocarcinoma	51	M
		Pleomorphic adenoma	Adenocarcinoma	66	M
		Pleomorphic adenoma	Salivary duct carcinoma	72	F
Azua-Romeo J^13^	2005	Oncocytoma	Acinic cell carcinoma	77	M
Yu G-Y^7^	2004	Warthin tumor	Squamous cell carcinoma	ND	ND
Zeegregts CJ^2^	2003	Pleomorphic adenoma	Acinic cell carcinoma	ND	ND
Shukla M^14^	2003	Sebaceous lymphadenoma	Squamous cell carcinoma	68	F
Curry JL^15^	2002	Pleomorphic adenoma	Salivary duct carcinoma	67	F
		Warthin tumor	Mucoepidermoid carcinoma	51	F
Mayorga M^16^	1999	Sebaceus lymphadenoma	Acinic cell carcinoma	78	F
Misselevich I^17^	1997	Pleomorphic adenoma	Acinic cell carcinoma	44	F
Seifert G^18^	1997	Warthin tumor	Mucoepidermoid carcinoma	73	M
Hanada T^19^	1995	Myoepithelioma	Adenoid cystic carcinoma	71	F
Gnepp DR^3^	1989	Warthin tumor	Mucoepidermoid carcinoma	60	M
		Warthin tumor	Acinic cell carcinoma	84	M
		Warthin tumor	Acinic cell carcinoma	56	M
		Warthin tumor	Ductal adenocarcinoma	69	M
		Warthin tumor	Adenoid cystic carcinoma	66	M
Janecka IP^20^	1983	Pleomorphic adenoma	Mucoepidermoid carcinoma	45	F
		Warthin tumor	Adenocarcinoma	64	M
		Warthin tumor	Mucoepidermoid carcinoma	58	M
Volmer J^21^	1982	Warthin tumor	Squamous cell carcinoma	85	F
Pontilena N^22^	1979	Pleomorphic adenoma	Mucoepidermoid carcinoma	45	F
Bab IA^23^	1979	Sebaceous cell adenoma	Adenoid cystic carcinoma	6th decade	F
		Oncocytoma	Carcinoma ex pleomorphic adenoma	6th decade	F
Bird RJ^24^	1979	Warthin tumor	Acinic cell carcinoma	ND	ND
Gadient SE^25^	1975	Warthin tumor	Mucoepidermoid carcinoma	60	M
Iannaccone P^26^	1975	Warthin tumor	Mucoepidermoid carcinoma	70	F
Lumerman H^27^	1975	Warthin tumor	Mucoepidermoid carcinoma	65	M
Turnbul AD^28^	1969	Warthin tumor	Carcinoma ex pleomorphic adenoma	ND	ND
		Pleomorphic adenoma	Adenocarcinoma	ND	ND
Tanaka N^5^	1953	Warthin tumor	Mucoepidermoid carcinoma	ND	ND

ND, no data.

This paper is the first one to present an in-depth study of a rare case of Warthin tumor coexisting with carcinoma ex pleomorphic adenoma in the same salivary gland. Only one occurrence of this kind has been just sparingly mentioned, without any study or analysis, in the English language literature in the 1960s.^12^, ^28^ The occurrence of carcinoma ex pleomorphic adenoma in our patient is typical for this type of malignancy (6th–7th decade of life), which may suggest that the synchronous concomitance of Warthin tumor was coincidental, and the initial state was the most popular combination of histologically different tumors – pleomorphic adenoma and Warthin tumor. The etiology of carcinoma ex pleomorphic adenoma is associated with the accumulation of genetic instabilities in long-standing pleomorphic adenomas, hence in the present case, the primary tumor was present for many years, what is important for process of malignization.^1^, ^6^, ^7^ Although there are some data about the incidence of multiple parotid tumors after radiotherapy, our patient had no history of radiation before.^12^

Clinical examination, imaging investigation and fine needle biopsy proved inefficient in this case. Although no particular type of radiological investigation has been defined in the detection of unilateral parotid tumors, the combination of ultrasound and MRI seems to have the best effectiveness rates in differentiating malignant lesions from benign ones.^29^ Fine needle aspiration cytology is crucial in the evaluation of parotid tumors. However, its role in case of unilateral synchronous tumor is controversial.^30^

Previous studies implied that the treatment and anticipated survival rate should be analogous to the cases of malignant neoplasms of the same histological type. Surgery is the gold standard in treatment of this kind of lesions. The presence of a malignancy might require a more aggressive approach, hence, depending on the nature and the location of the tumor – total or subtotal parotidectomy is indicated.^6^ Intraoperative frozen section biopsy might add important information that could alter the management and improve the final outcome of the treatment. Our case seems to confirm that a routine use of this examination may have influenced the extent of the surgical management in the way that the total parotidectomy and the elective I and II level lymph node removal would be performed. Thus, re-operation would not be necessary.^31^

Adjuvant radiotherapy is highly recommended for high-grade malignancies like carcinoma ex pleomorphic adenoma, due to a high risk of locoregional recurrence. Due to the fact that the surgical treatment was not optimal in this case, the patient was qualified for radiation therapy in order to minimize the risk of subclinical microscopic spread of the disease.^32^

## Conclusions

Multiple synchronous unilateral parotid tumors may cause significant discrepancies between the preliminary and definitive histopathological prognosis, especially when pre-operative clinical assessment and FNAC did not indicate the presence of two different neoplasms within one gland. The awareness of the coexistence of benign and malignant lesions in ipsilateral parotid gland should raise the clinical vigilance in the process of evaluation of a parotid mass.

## Conflicts of interest

The authors declare no conflicts of interest.
